# Conversion of embryonic stem cells into extraembryonic lineages by CRISPR-mediated activators

**DOI:** 10.1038/srep19648

**Published:** 2016-01-19

**Authors:** Shu Wei, Qingjian Zou, Sisi Lai, Quanjun Zhang, Li Li, Quanmei Yan, Xiaoqing Zhou, Huilin Zhong, Liangxue Lai

**Affiliations:** 1 CAS Key Laboratory of Regenerative Biology, South China Institute for Stem Cell Biology and Regenerative Medicine, Guangzhou Institutes of Biomedicine and Health, Chinese Academy of Sciences, Guangzhou, 510530, China; 2 Guangdong Provincial Key Laboratory of Stem Cell and Regenerative Medicine, South China Institute for Stem Cell Biology and Regenerative Medicine, Guangzhou Institutes of Biomedicine and Health, Chinese Academy of Sciences, Guangzhou 510530, China; 3 Jilin Provincial Key Laboratory of Animal Embryo Engineering, Institute of Zoonosis, College of Veterinary Medicine, Jilin University, Changchun, 130062, China

## Abstract

The recently emerged CRISPR/Cas9 technique has opened a new perspective on readily editing specific genes. When combined with transcription activators, it can precisely manipulate endogenous gene expression. Here, we enhanced the expression of endogenous Cdx2 and Gata6 genes by CRISPR-mediated activators, thus mouse embryonic stem cells (ESCs) were directly converted into two extraembryonic lineages, i.e., typical trophoblast stem cells (TSCs) and extraembryonic endoderm cells (XENCs), which exhibited characters of TSC or XENC derived from the blastocyst extraembryonic lineages such as cell morphology, specific gene expression, and differentiation ability *in vitro* and *in vivo*. This study demonstrates that the cell fate can be effectively manipulated by directly activating of specific endogenous gene expression with CRISPR-mediated activator.

Enforced exogenous transcription factor expression can remodel cell fate, for instance, combined expression of pluripotent transcription factors, Oct4, Sox2, Klf4 and c-Myc can convert somatic cells into pluripotent cells (iPSCs)^1^,^2^. Ectopic expression of neural lineage-specific transcription factors, Brn2, Ascl1 and Myt1l can convert fibroblasts or hepatocytes into neurons^3^,^4^,^5^. Ectopic expression of lineage-specific transcription factors, Hnf4a, Gata4 and Foxa3 can transdifferentiate fibroblasts into hepatocytes^6^,^7^. However, the exogenous transcription factors may cause safety concerns and present a formidable obstacle to the therapeutic use of induced cells.

Recently, a site-specific nuclease clustered regularly interspaced short palindromic repeats (CRISPR)/CRISPR-associated (Cas) 9 system has been engineered to target and edit specific genome sites through a synthetic single-guide RNA (sgRNA) with simple base-pair complementarities^8^. Compared with the fixed DNA sequence-binding repetitive composition requirements of ZFNs and TALEs, the RNA-guide platform is fairly easy to master and practice. The CRISPR/Cas9 system has been used for genome editing in different organisms, including prokaryotes and eukaryotes^9^,^10^,^11^. Moreover, by fusing with a mutant Cas9 protein lacking endonuclease activity (dCas9) with transactivator VP64 or repressor KRAB, the fusion proteins can be guided to the desired promoter regions and modulate up- or down-regulation of endogenous gene expression^12^,^13^,^14^.

The mouse blastocyst (~3.5 days post coitum, d.p.c.) comprises three lineages i.e., pluripotent epiblasts and two extraembryonic tissues- [i.e., trophectoderm (TE) and primitive endoderm (PrE)]^15^. The three lineages can be cultured *in vitro*. Epiblast can be cultured as embryonic stem cells (ESCs); TEs can be converted to trophoblast stem cells (TSCs); PrE can form extraembryonic endoderm stem cells (XENCs)^16^. Previous studies have found that the two extraembryonic cells can be generated from embryonic stem cells by exogenous Cdx2^17^ or Gata6^18^ overexpression, respectively. In this study, we fused VP64 with dCas9, and designed sgRNAs to target the promoter regions of Cdx2 and Gata6. By co-transfection of the fused proteins and sgRNAs, we succeeded in activating endogenous Cdx2 and Gata6 in mESCs, thus converting ESCs into TSCs and XENCs, respectively. This study provides an effective approach to manipulate cell fate by directly activating specific endogenous genes using only CRISPR-mediated activator system. This technique can be used to study other biological processes such as cell reprogramming or transdifferentiation.

## Results

### Generation of Cdx2 reporter in ES cells

To conveniently trace Cdx2 expression, we constructed a Cdx2-2A-tdTomato (CT) reporter system in OG2 mESCs, which carry an Oct4 promoter-driven EGFP transgene. A double-stranded donor vector was designed to insert a P2A-tdTomato reporter into the last codon of the Cdx2 gene. Cas9, sgCdx2 (sgRNA targeting around Cdx2 termination codon), and the donor were co-transfected into OG2 mESCs (Supplementary Fig. 1a). After 7 days, the ESC colonies were picked for PCR identification, and the colonies with 2A-tdTomato knock-in were named OG-CT mESCs (Supplementary Fig. 1b). To verify the reliability of this system, OG-CT mESCs were injected into enucleated oocytes. After 4 days, the reconstructed embryos developed into blastocysts with EGFP expression in the inner cell masses (ICMs) and tdTomato expression in the surrounding TE cells (Supplementary Fig. 1c). Thus, the OG-CT mESCs were suitable for tracing both Oct4 and Cdx2 expressions.

### Activation of endogenous Cdx2 by CRISPR-mediated activator

**Figure 1:**
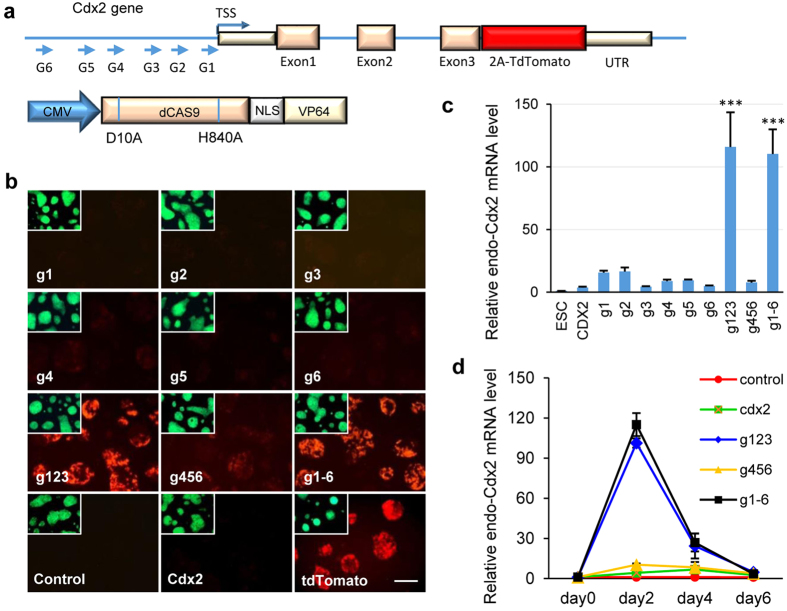
Endogenous Cdx2 expression activation in ESCs. (**a**) Schematic dCas9-VP64 vector design and 6 sgRNAs (g1 to g6) targeted to the promoter of endogenous mouse Cdx2 gene. 2A-tdTomato gene had been precisely knocked into the last exon of Cdx2 gene. (**b**) tdTomato activation in OG-CT ESCs after transfecting with multiplex combination of C9V64/sgRNAs, exogenous Cdx2 or exogenous tdTomato for 2 days. EGFP expression was shown on the upper left corner of each picture. Scale bar, 50 μm. (**c**) Q-PCR analysis of endogenous Cdx2 mRNA level after transfecting different activators into OG-CT ESCs for 2 days. Gene expression values are normalized to the expression of Gapdh. (Mean ± s.d., two-tailed, unpaired t-test; n = 3 independent experiments, ^***^p < 0.001). (**d**) Quantitation of endogenous Cdx2 mRNA level after transfecting different activators into OG-CT ESCs at various time points. Gene expression values are normalized to the expression of Gapdh. (*n* = *3*).

To create the CRISPR-mediated activator system, we genetically fused dCas9 (D10A and H840A) with C-terminal VP64 acidic transactivation domain (dCas9-VP64, C9V64 for short) as previously reported^13^. Meanwhile, we identified six sgRNA target sites from 500 bp to 50 bp upstream of Cdx2 transcription start site (TSS) (Fig. 1a). Synthetic sgRNAs g1 to g6 were introduced into sgRNA scaffold vectors (pX330) under the control of hU6 promoter. The sgRNA plasmids were subsequently co-transfected with C9V64 into OG-CT mESCs. After 48 h, high-level red fluorescence was observed in groups g123 (g1, g2, g3 co-transfected with C9V64) and g1 ~ 6 (all 6 sgRNAs co-transfected with C9V64). However, the tdTomato activity was hardly detected in other groups, such as single sgRNA group (with C9V64), g456 group (with C9V64), control group (without sgRNAs), and exogenous Cdx2 group (Fig. 1b), indicating that the combination and target sites of sgRNAs are critical for endogenous gene activation^19^. Quantitative PCR (Q-PCR) analysis revealed that endogenous Cdx2 expression levels were boosted more than 100-fold in groups g123 and g1 ~ 6 (Fig. 1c), confirming that the endogenous Cdx2 expression was consistent with tdTomato activity. Given that the exogenous sgRNA and activator are only transiently expressed in ESCs, we further tested the expression level of endogenous Cdx2 after CRISPR-mediated activator transfection. In groups g123 and g1 ~ 6, endogenous Cdx2 mRNA expressed the highest level on day 2 and rapidly decreased thereafter (Fig. 1d).

### Conversion of ESCs into TSCs by directed activation of endogenous Cdx2

**Figure 2:**
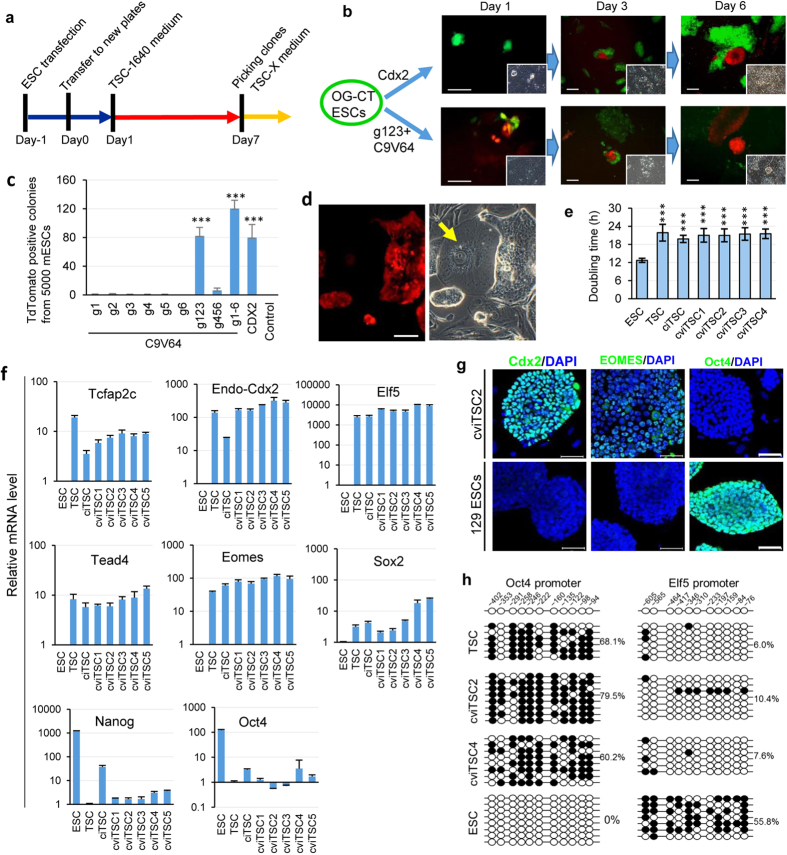
Conversion of ESCs into TSCs by activation of endogenous Cdx2. (**a**) Timeline of converting ESCs into TSCs by CRISPR/Cas9-activator system. (**b**) tdTomato expression and TSC-like colonies formation after 1, 3 and 6 days of exogenous Cdx2 or C9V64/g123 delivered into OG-CT mESCs. Phase contrast were shown one the lower right corner of each picture. Scale bar, 50 μm. (**c**) Quantification of TSC-like colonies labeled with red fluorescence from OG-CT ESCs with different combinations of activators (C9V64 plus gRNAs), exogenous Cdx2 and control group (pCDNA3.1). Mean ± s.d., two-tailed, unpaired t-test; n = 3 independent experiments, ^***^p < 0.001. (**d**) Typical TSC-like colonies (named cviTSCs) with uniform red fluorescence after 10 passages. Arrow points the spontaneous differentiated TGC. Scale bar, 50 μm. (**e**) Doubling time of ESCs, TSCs and cviTSCs. (mean ± s.d., two-tailed, unpaired t-test; n = 4 independent experiments, ^***^p < 0.001). (F) Q-PCR analysis of the mRNA level of TSC lineage markers (Tcfap2c, endogenous Cdx2, Elf5, Tead4, Eomes and Sox2) and ESC lineage markers (Oct4 and Nanog) in cviTSCs. (*n* = *3*). (**g**) Immunostaining of Cdx2, Eomes and Oct4 in cviTSCs and ESCs. cviTSCs express TSC markers but not ESC marker. Scale bar, 50 μm. (**h**) Methylation status of differentially methylated regions in the promoters of Oct4 and Elf5 in TSCs, cviTSCs, and ESCs. Blank and filled circles represent unmethylated and methylated CpG islands, respectively.

We subsequently transfected a single Cdx2 expression vector and C9V64/sgRNAs into OG-CT mESCs, respectively. These cells were passaged to new plates the following day (day 0). The next day, the medium was changed to TSC-1640 medium containing fetal bovine serum (FBS) and FGF4 until formation of TSC-like colonies (Fig. 2a). Upon exogenous Cdx2 overexpression in OG-CT mESCs, the red fluorescence started to emerge, and the green fluorescence simultaneously disappeared in some cells on day 3, thus indicating endogenous Cdx2 activation. After another 3 days, these cells proliferated to tight and epithelial sheet like colonies with smooth expanding margins (Fig. 2b, above). When C9V64 and g123 were delivered into OG-CT mESCs, as expected, red fluorescence emerged rapidly and co-expressed with green fluorescence on day 1. Eventually, green fluorescence faded, and colonies with merely red fluorescence formed on day 3. These colonies also expanded to epithelial sheets-like colonies on day 6 (Fig. 2b, down).

Thereafter, we examined the conversion efficiency of the different groups and found that g123 group and g1 ~ 6 group showed high efficiency (~2%) (Fig. 2c), indicating that direct activation of endogenous Cdx2 genes can efficiently regulate cell fate. On day 7, the colonies were retrieved and passaged to new plates pre-coated with Matrigel and feeder cells and were cultured in serum-free TSC-X medium with FGF4 and TGF-beta^20^. After 2–3 passages, the converted cells formed standard TSC-like colonies with uniform red fluorescence (Fig. 2d). The typical TSC phenotype can maintain for more than 30 passages. We named the exogenous Cdx2-induced cells as ciTSCs, and the C9V64/g123-induced cells as cviTSCs. The cviTSCs were further injected into enucleated oocytes. Similar to that of OG-CT ESCs, the reconstructed embryos also developed into blastocysts with EGFP expression in ICMs and tdTomato expression in surrounding TE cells (Supplementary Fig. 1d), confirming that the cviTSCs originated from OG-CT ESCs.

### Characterization of cviTSCs

Compared with original ESCs, cviTSCs proliferated relatively slow. The doubling time (19–22 h) was similar to ciTSCs and TSCs de novo derived from blastocysts (Fig. 2e). To further investigate the gene regulation pattern induced by CRISPR-mediated activator, several pluripotent and trophoblast markers were examined by Q-PCR. Tcfap2c, Cdx2, Elf5, Tead4, Eomes and Sox2 are the indispensable characteristic transcription factors for maintaining TSC stemness^21^. Herein, the cviTSCs exhibited the same activation level of these factors as that of ciTSCs and TSCs (Fig. 2f, first two rows). Conversely, both pluripotent factors (Oct4 and Nanog) were significantly down-regulated in cviTSCs (Fig. 2f, last row). Consistent with the mRNA expression pattern, immunostaining assay demonstrated that cviTSCs expressed Cdx2 and Eomes, but not Oct4, in contrast with mESCs (Fig. 2g). Stable lineage conversion should be accompanied by epigenetic remodeling. Bisulfite sequencing was used herein to assess the CpG island methylation status of cviTSC Oct4 and Elf5 promoters, comparing them with TSCs and ESCs^22^. Oct4 promoter elements were highly methylated in TSCs (greater than 60%) and completely demethylated in ESCs (0%). By contrast, the promoter of the trophoblast marker Elf5 was highly methylated in ESCs (55.8%) and largely demethylated in TSCs (approximately 10%, Fig. 2h). The two promoter elements of cviTSCs displayed a methylation status similar to TSCs, suggesting that the epigenetic markers of ESC-lineage had been replaced by the TE markers.

### Differentiation of cviTSCs *in vitro* and *in vivo*

**Figure 3:**
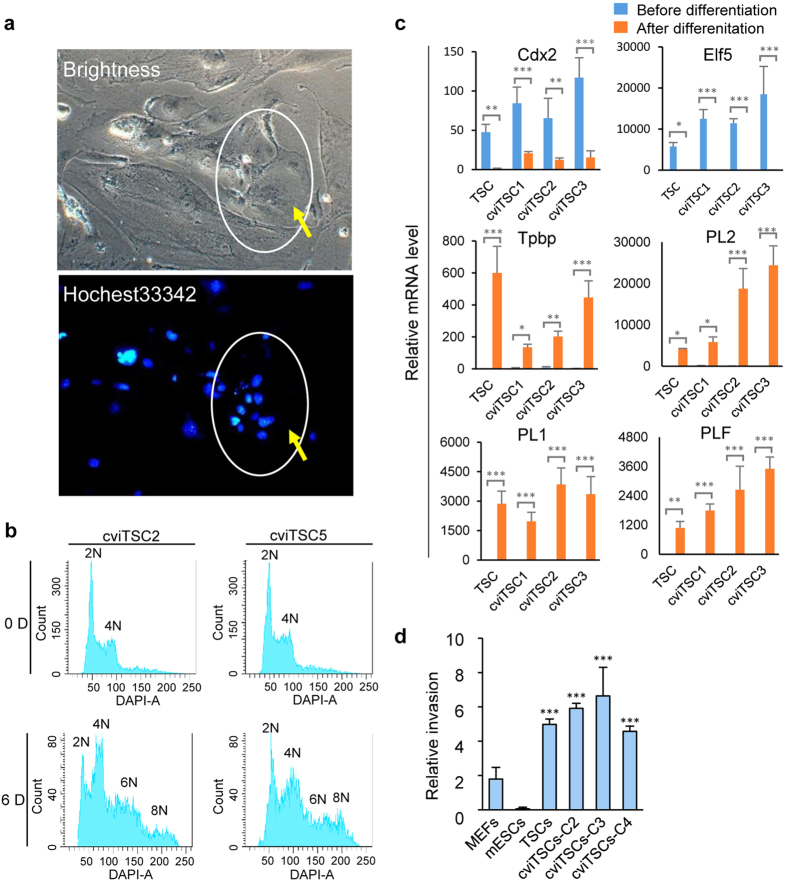
Differentiation of cviTSCs *in vitro*. (**a**) Nuclear staining of multi-nucleated trophoblast cells after 7 days in differentiation culture. Nuclear were stained by Hochest33342. Yellow arrows pointed the multi-nucleated trophoblast cells. Scale bar, 200 μm. (**b**) DNA content analysis of cviTSCs before and after differentiation by FACS. (**c**) Q-PCR analysis of the mRNA level of TSC lineage markers (endogenous Cdx2, Elf5), spongiotrophoblast marker (Tpbp), and terminally differentiated TGC markers (PL1, PL2 and Plf) before and after cviTSC differentiation. Gene expression values are normalized to the expression of Gapdh. (Mean ± s.d., two-tailed, unpaired t-test; n =  3 independent experiments, ^*^p < 0.05, ^**^p < 0.01, ^***^p < 0.001). (**d**) The comparison of the invasive ability of cviTSCs, TSCs and ESCs on Matrigel coated transwell plates. (mean ± s.d., two-tailed, unpaired t-test; n = 3 independent experiments, ^***^p < 0.001).

cviTSCs and TSCs grew for more than 10 passages in TSC-X medium before being conducted for differentiating. Cells were subsequently plated in feeder-free TSC-1640 medium without growth factor FGF4 for 6 days. cviTSCs stopped proliferating and eventually differentiated, becoming indistinguishable flat and large (Fig. 3a). The enlarged cells demonstrated to have 1 to more than 10 nuclei per cell. The cells with 1–2 nuclei were probably trophoblast giant cells (TGCs), and the cells with 3 or more nuclei might be syncytiotrophoblast (synT)^23^. The nuclear DNA content of cviTSCs and the differentiated cells were quantified by flow cytometry (FCM) analysis. Consistent with nuclear number, cviTSCs showed only diploid and tetraploid DNA content, meanwhile differentiated cells demonstrated polyploid DNA content (Fig. 3b). Both cviTSCs and TSCs were significantly downregulated of Cdx2 and Elf5 after differentiation, whereas markers for intermediate differentiated stage of spongiotrophoblast (Tpbp) and terminally differentiated TGC (PL1, PL2 and Plf) were strongly upregulated (Fig. 3c). To verify the TGC invasive ability, Matrigel-coated transwell was used to mimic the three-dimensional structure of the endometrium, allowing assessment of the invasive capacity of TGCs *in vitro*^24^. In our invasive assay, about 4–8% (n = 3) of differentiated cviTSCs and TSCs invaded the transwell and attached to the bottom of the membrane, whereas ESCs had less than 0.2% invasion rate (Fig. 3d).

**Figure 4:**
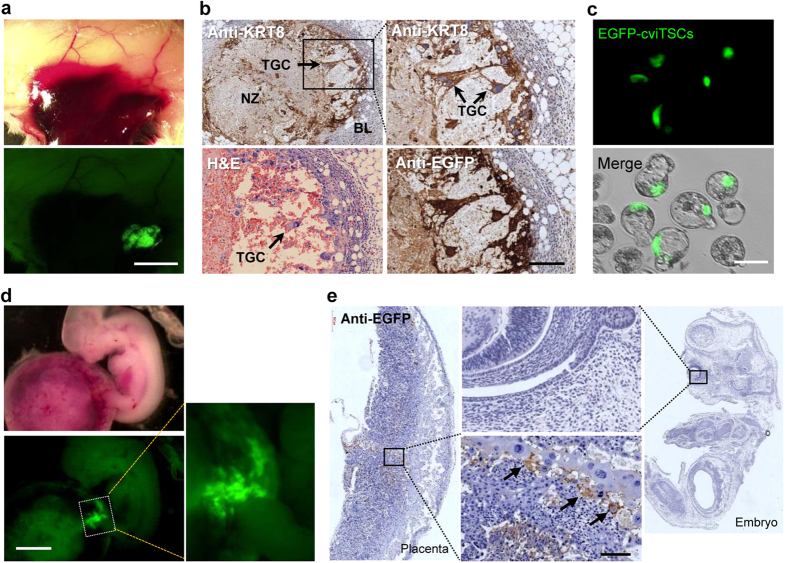
Differentiation of cviTSCs *in vivo.* (**a**) Formation of blood-filled sinuses under murine skin 6 days after subcutaneous injection of EGFP labeled cviTSCs. Scale bar, 5 mm. (**b**) Hematoxylin and eosin and immunohistochemistry (IHC) analysis of the blood-filled-sinuses. EGFP and KRT8 were stained in brown in the same cells with large nuclear. TGC: trophoblast giant cell; NZ: necrosis zone; BL: basal lamina, Scale bar, 100 μm. (**c**) Blastocysts integrated with EGFP-cviTSCs. cviTSCs were injected into 4-cell-stage embryos and integrated into trophectoderm after the blastocyst formation. Scale bar, 100 μm. (**d**) Chimeric placenta following blastocyst injection of EGFP-cviTSCs. cviTSCs contributed to the placenta, but not to the fetus. Scale bar, 5 mm. (**e**) IHC analysis of the chimeric placenta. EGFP positive cviTSC-derived cells were embedded within the placenta (black arrows), but not in the fetus. Scale bar, 200 μm.

To investigate the *in vivo* differentiation potential, cviTSCs were labeled with EGFP and injected subcutaneously into the flank of ICR mice. A week after injection, solid tumors with blood-filled sinuses formed subcutaneously (Fig. 4a, up). EGFP-positive cells were integrated in the tumors (Fig. 4a, below). Tumor sections were stained with hematoxylin and eosin (H&E) to examine the histological structure. Cells with large nucleus were observed in the tumors. Immunohistochemistry (IHC) staining was conducted to further test EGFP and placenta marker KRT8 expression. EGFP and KRT8 were co-expressed in the same cells with large nucleus, indicating that these cells were exclusively derived from cviTSCs, rather than embryonic germ layer derivatives (Fig. 4b). This finding also indicates that cviTSCs differentiated into TGCs with invasive capability into host vessels^25^.

Subsequently, EGFP-cviTSCs were injected into 4-cell-stage embryos to create chimeric embryos. After the embryos developed to blastocyst stage, green fluorescence could be detected in the TE (Fig. 4c). Thereafter, blastocysts were injected with the same cviTSCs and transferred into uterus of surrogate mice. After 9 days, the mice were sacrificed, both fetus and placenta were collected and observed under fluorescent microscope. The GFP-positive cells showed an exclusively placental contribution (Fig. 4d) as that of the embryo-derived TSCs^26^. Immunohistochemical analysis showed that the EGFP-positive grafts were incorporated into the normal tissue architecture of the placenta, but not embedded into the fetus portion (Fig. 4e and Supplementary Fig. 2), confirming their ability to function as TSCs.

### Generation and characterization of cviXENCs

**Figure 5:**
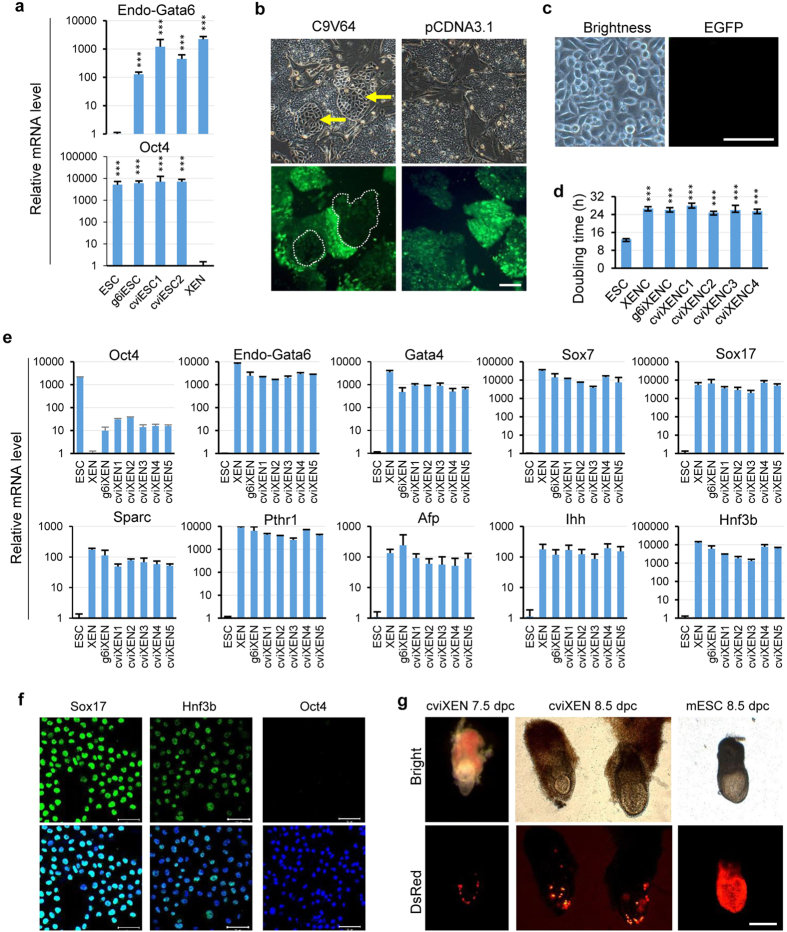
Generation and characterization of cviXENCs by activation of endogenous Gata6. (**a**) Q-PCR analysis of endogenous Gata6 and Oct4 mRNA level after transfecting CRISPR-mediated activators into OG2 ESCs for 3 days. Gene expression values were normalized to the expression of Gapdh. (mean ± s.d., two-tailed, unpaired t-test; n = 3 independent experiments, ^***^p < 0.001). (**b**) Epithelia-like colony formation after 5 days of C9V64/sgRNA (Gata6) delivered into OG2 mESCs. EGFP was still active in the epithelia-like colonies. No epithelia-like colonies were found in the control group (pCDNA3.1). Scale bar, 50 μm. (**c**) Typical XEN-like colonies (named cviXENCs) after culture for 10 passages. EGFP expression was silenced in cviXENCs. Scale bar, 50 μm. (**d**) Doubling time of ESCs, XENCs, g6iXENCs, and cviXENCs. (mean ± s.d., two-tailed, unpaired t-test; n = 3 independent experiments, ^***^p < 0.001). (**e**) Q-PCR analysis of the mRNA level of XEN lineage markers (Gata4, Gata6, Sox7 and Sox17), VE markers (Sparc, Pthr1 and Afp), PE markers (Ihh and Hnf3b), and ESC lineage markers (Oct4) in cviXENCs. VE, visceral endoderm; PE, parietal endoderm. (*n* = *3*). (**f**) Immunostaining of Sox17, Hnf3b, and Oct4 in cviXENCs. cviXENCs expressed XENC markers Sox17 and Hnf3b but not ESC marker Oct4. Scale bar, 50 μm. (**g**) The 7.5 and 8.5 d.p.c. chimeric embryos following blastocyst injection of DsRed-cviXENCs. cviXENCs contributed exclusively to the distal parietal yolk sac, but not to the embryo. DsRed-positive mESCs were integrated into the whole embryonic portion. Scale bar, 500 μm.

The process of activating endogenous Gata6 in mESCs was similar to that of endogenous Cdx2. Two sgRNA target sites were set on the upstream of Gata6 TSS. Vectors containing both sgRNA and C9V64 were linearized and then transfected into OG2 mESCs. Culture medium was replaced with fresh XEN medium the following day. Three days after the initial transfection, we collected the cells for Q-PCR test. As shown in Fig. 5, endogenous Gata6 expression in the cells was boosted about 1000-fold (Fig. 5a, up), nevertheless Oct4 expression still remained (Fig. 5a, down). Epithelial-like cell colonies surrounded with undifferentiated mESCs appeared 4–5 days after transfection (Fig. 5b). A low expression of EGFP protein still existed in the colonies. No epithelial-like cells emerged in the control group. On day 6, epithelial-like colonies were retrieved and seeded to new plates pre-coated with Matrigel. After 3–4 passages, the uniform XEN-like cells with stellate and refractive morphology formed and EGFP expression had not been observed in cviXENCs. (Fig. 5c).

We named the sgRNA/CRISPR induced cells as cviXENCs, the exogenous Gata6 induced cells as g6iXENCs, and blastocysts-derived cells as XENCs. Compared with ESCs and TSCs, the cviXENCs proliferated relatively slow. The doubling time (24 ~ 27 h) of cviXENCs was similar to g6iXENCs and XENCs (Fig. 5d). To investigate the characteristics of cviXENCs, we analyzed marker gene expression in passage 12 cells by Q-PCR. We found that Gata4, Gata6, Sox7 and Sox17, which are markers of XENCs, were highly expressed in cviXENCs and g6XENCs as that in XENCs (Fig. 5e). *In vivo*, PrE subsequently differentiates into visceral endoderm (VE) and parietal endoderm (PE)^27^. To investigate whether the cviXENCs express PE and VE markers, we further analyzed the expression level of the PE markers Sparc and Pthr1, and the VE markers Afp, Ihh and Hnf3b. We also found that all these markers were activated in cviXENCs and g6XENCs at higher level as that of XENCs (Fig. 5e). By contrast, the pluripotent marker Oct4 was significantly downregulated in cviXENCs. Consistent with the mRNA expression pattern, immunostaining assay demonstrated that cviXENCs expressed Sox17 and Hnf3b, but not Oct4 (Fig. 5f). To test the differentiation potential of cviXENCs, these cells were labeled with DsRed and subsequently injected into wild-type ICR blastocysts (3.5 d.p.c.) and transferred into the uteri of pseudopregnant mice. After 4 and 5 days, mice were sacrificed, and the reconstructed embryos (7.5 and 8.5 d.p.c) were collected and observed under fluorescent microscope. The DsRed-positive cells contributed exclusively to the extraembryonic yolk sac with a scattered pattern in the chimeric embryos (Fig. 5g). By contrast, DsRed-positive mESCs were integrated into the whole embryonic portion. It indicates that cviXENCs derived from ESCs by artificial activation of endogenous Gata6 have the potential to contribute to only PE *in vivo*.

## Discussion

The CRISPR-mediated activator system has been used for the activation of specific endogenous genes, and is beneficial for the study of gene function, gene therapy, and genetic reprogramming^12^,^13^. In addition, CRISPR-mediated activator system can also be used to manipulate the expression of endogenous non-coding RNAs that exist in untranslated regions and play important roles in cell fate regulation. Through the direct control of endogenous specific gene expression, CRISPR-mediated activator system provides a novel, low-cost and promising method for reprogramming cell lineage specification.

Chakraborty *et al.* converted fibroblasts into myoblasts by activating endogenous MyoD gene^28^; Chavez *et al.* promoted the efficiency of ESCs differentiate into neurons by activating endogenous Ngn2 or NeuroD1^29^. The complex activators, two VP64 domains flanking dCas9 and a hybrid VP64-p65-Rta tripartite activator were used respectively in those reports. In this study, we first showed that C-terminal fusion of a single VP64 to dCas9 is sufficient to robustly activate endogenous Cdx2 and Gata6 in ESC, and resulted in high efficiency of TSC and XENC induction. Previous report has shown that a peak of active sgRNAs for CRISPR-mediated activation is located at −400 to −50 bp upstream from the TSS^30^. For activation of Cdx2 and Gata6, we further found that the most active sgRNA target sites were in the range of −200 to −40 bp upstream from TSS.

The Cdx2 is robustly expressed in TE, the first differentiated cell lineage of mammalian embryogenesis, which forms the placenta, a structure unique to mammalian development^17^. Oct4 is another central lineage player that is required for maturation of blastocyst ICM^31^. The two lineage marker genes exhibit reciprocal inhibition by binding to each other’s promoters^17^. Ectopic expression of Cdx2 triggers ESCs conversion to the TE lineage, whereas overexpression of Oct4 converts TSCs into ESCs^32^. The expression level of Cdx2 is critical for converting ES cells into TSCs. High endogenous gene expression level can be realized by modification of activator composition^28^,^29^, or application of multiple sgRNAs with optimized position^33^. In our study, we employed the second strategy, i.e., transfection of 3 optimized sgRNAs (g123) with C9V64. The dual-gene reporter system combined with quantitative Q-PCR analysis showed that endogenous Cdx2 expression levels were boosted more than 100-fold in group g123 two days after transfection, while Oct4 expression was soon stopped. The two events (activation of Cdx2 and silence of Oct4) promoted the conversion of ESCs into TSCs.

Gata6 is the key transcription factor in PrE formation during early embryogenesis, and also localizes to endo- and mesodermal cells during later embryogenesis^34^. Ectopic expression of each GATA6 or other PrE lineage marker gene, such as Gata4 or Sox17 is sufficient to convert ESCs into XENCs^18^,^35^. In this study, we also used the strategy of a simple activator (C9V64) combining multiple sgRNAs to activate endogenous GATA6 in ESCs. The expression level increased by more than 1000 times in 3 days after transfected with C9V64 plus 2 sgRNAs, which was sufficient to initiate the conversion of ESCs into XENCs, and able to result in XEN cell lines with long term passage capacity. Gata6 does not directly repress the expression of Oct4 as that of Cdx2. Thus, Oct4 still expressed when epithelial-like cells formed. After 3 to 4 passages, EGFP expression in epithelial-like cells was silenced, indicating that the silence of endogenous Oct4 is a slow and gradual process.

Both induced extraembryonic lineages (cviTSC and cviXENC) generated by directly activating endogenous extraembryonic lineage markers (Cdx2 and Gata6), exhibited typical characters of extraembryonic lineages derived from the blastocysts, such as cell morphology, proliferation, epigenetic state, lineage specific gene expression, and differentiation ability *in vitro* and *in vivo*.

In this study, we proved that activation of endogenous Cdx2 and Gata6 could be effectively realized by fusion of a single VP64, the most commonly used activator, with dCas9. However, this system may not be all that effective for other genes. Many efforts tried to activate endogenous transcription factors using single VP64-based activators, but failed to achieve sufficient expression level to initiate conversion of cell fate^29^,^33^,^36^,^37^. Thus far, a universal activator which is able to activate a variety of genes has not been available yet. Therefore, a specific activation strategy needs to be designed for an individual gene. Other activation domains containing recruiting multiple components of the pre-initiation complexes such as P65AD and RTA, or the catalytic histone acetyltransferase core domain of the human E1A-associated protein p300^29^,^38^ have been employed to improve the efficiency of endogenous gene expression. The optimization of components of transcription activators, number and locus of sgRNAs and epigenetic editing factors is necessary to make an efficient activation strategy for a specific gene.

## Methods

### Ethics statement

The animal experiment facilities were approved by the Department of Science and Technology of Guangdong Province (ID SYXK 2005-0063). Surgical procedures were performed under anesthesia in accordance with guidelines of Institutional Animal Care and Use Committee of Guangzhou Institute of Biomedicine and Health, Chinese Academy of Sciences (Animal Welfare Assurance #A5748-01), and all efforts were made to minimize suffering.

### Cell culture

All cell culture reagents were purchased from Life Technology except the specially maintained reagents.

Mouse ESC lines were derived from OG2 mice, which carry an Oct4 promoter-driven EGFP transgene for tracing the naïve pluripotent cells^39^. Mouse ESCs were cultured on mitomycin C-treated mouse embryonic fibroblast (MEF) feeder layers with ES medium containing Dulbecco’s Modified Eagle’s Medium (DMEM), 15% fetal bovine serum (FBS), 2 mM GlutaMax, 1% nonessential amino acids, 0.1 mM β-mercaptoethanol, 1,000 units/mL ESGRO (Millpore), 1 mM PD0325901 and 3 mM GSK3 inhibitor CHIR99021.

TSCs were derived as previously reported^20^ with slight modifications. Briefly, Blastocysts were collected from the ICR females after mating for 3.5 days. The blastocysts were cultured on mitomycin C-treated mouse embryonic fibroblast (MEF) feeder layers with TSC-1640 medium containing RPMI 1640, 20% FBS, 1 mM sodium pyruvate, 2 mM GlutaMax, 0.1 mM β-mercaptoethanol, 25 ng/mL FGF4 (PeproTech), and 1 μg/mL heparin (Sigma). Blastocysts attached to the feeder layers within 2 ~ 3 days. After another 4 days, the blastocyst clumps were disaggregated in 0.05% trypsin-EDTA and subsequently plated on new feeder layers. One week later, TS colonies were found in epithelial sheets with tight boundary. The colonies were passaged and cultured in TSC-1640 medium or serum-free medium, named TSC-X medium, containing with DMEM/F12, 2 mM GlutaMax, 2% Insulin-Transferrin-Selenium (ITS-G), 50 mg/L l-ascorbic acid-2-phosphate magnesium (Sigma), 25 ng/mL human FGF4 (PeproTech), 1 μg/mL heparin (Sigma), and 2 ng/mL human recombinant TGF-ß1 (PeproTech). When TSCs were cultured in TSC-X medium, the plates need pre-coated with 2% (v/v) Matrigel (BD).

XENCs were derived as previously reported^40^, and cultured in Matrigel coated dishes in XEN medium containing RPMI 1640, supplemented with 15% (vol/vol) FBS, 2 mM GlutaMax and 0.1 mM β-mercaptoethanol.

### Vector construction

To construct pCMV-Cdx2 and pCMV-Gata6 vector, Cdx2 and Gata6 genes were amplified by RT-PCR, respectively, followed by cloning into pEGFP-N2 vector (Clontech) between EcoRI and NotI. To construct pCMV-dCAS9-VP64 vector, hCAS9 vector (#41815, Addgene, a gift from George Church) was mutated (D10A + H840A) and named dCAS9, and VP64 activation domain module was fused with the dCAS9 gene as previously described^41^. U6-sgRNA cloning vector was also a gift from George Church (#41819, Addgene). sgRNAs were designed by GN19NGG rule and were constructed as previously described^10^. The primer sequences for sgRNAs that guide dCas9 to Cdx2 promoter (sgCdx2P) and Gata6 promoter (sgGata6P) loci were designed as shown in Supplementary Table 1.

To generate a Cdx2-reproter system, sgCdx2 was designed as: caccGGCGGCGGCACAGCAATCCC and aaacGGGATTGCTGTGCCGCCGCC. U6-sgCdx2 was amplified and cloned into hCAS9 vector between MfeI and SpeI. Left homology arm (HA-L) and right homology arm (HA-R) around the Cdx2 stop codon site amplified from mouse ESC genome by PCR were cloned into upstream and downstream of 2A-tdTomato, as described in Supplementary Fig. 1a.

### Generation of Cdx2-td Tomato reporter system

U6-sgCdx2-hCAS9 and donor vectors in ratio of 1:3 (w/w) were transfected into OG2 ESCs using Xfect™ mESC Transfection Reagent (Clontech) as instructed. U6-sgCdx2-hCAS9 contained a Neomycin gene (neo), conferring G418 resistant ability to the transfected cells. ESC medium was refreshed everyday with 800 μg/mL G418 (Sigma) for one week. G418 resistant colonies were retrieved for further culture. Portion of each colonies was collected for genomic PCR to detect 2A-td Tomato insertion. The primers are ROPG-F: 5′-TTGATTTATGGAAAGGAGGGGTGC-3′ and ROPG-R: 5′-GTGGAGGTGGGAGTGGGAAGATAATG-3′.

### Activation of endogenous Cdx2 and Gata6 in mESCs

pCMV-dCAS9-VP64 vector (C9V64) and sgCdx2Ps vector were co-transfected into OG2 mESCs using Xfect™ mESC Transfection Reagent (Clontech) as mentioned above. pcDNA3.1 and pCMV-Cdx2 vector were used as negative and positive control, respectively. The following day, the ESCs were dissociated into single cells and seeded to 1 × 10^4^ on 35 mm tissue culture dishes with feeder layer. After another day, the culture medium was refreshed with TSC-1640 medium, and performed every other day. After TSC colony formation, the medium was changed to TSC-X medium. Endogenous Gata6 was activated the same way with XEN medium replaced 1 day after transfection.

### ESC and TSC nuclear transfer

The main process of nuclear transfer was similar to a previous report^42^. B6D2F1 female mice (C57BL/6 × DBA/2) were superovulated with 7 U of pregnant mare’s serum gonadotropin for 48 h and 9 U of human chorionic gonadotropin for 14 h. Metaphase II (MII) oocytes were subsequently flushed from the uteri. The spindle-chromosome complexes of MII oocytes were enucleated using a blunt Piezo-driven pipette in a droplet of HEPES-CZB medium containing 5 μg/mL cytochalasin B (CB) and followed by maintained in CZB medium. 50 EGFP positive ESCs and 45 tdTomato positive TSCs were injected into the enucleated oocytes using a micromanipulator, respectively. Reconstructed oocytes were cultured in CZB medium for approximately 1 ~ 3 h before activation. Thereafter, activation was achieved for 6 h in calcium-free CZB medium containing 10 mM of strontium chloride and 5 μg/mL of CB. The reconstructed embryos were further cultured in G1 medium for the next 4 h, and subsequently cultured in G1 and G2 medium (Vitrolife) at 37 °C under 5% CO_2_ for 3.5 days to form blastocysts.

### Cell Proliferation Assay

Cells were plated at 40,000 per well of 12- well plate, and cultured with growth medium. After 4 days, cells were dissociated in trypsin-EDTA (0.05%, Life Technologies). Cell number was counted by Scepter™ 2.0 Cell Counter (Millipore).

### RNA isolation and real-time PCR analyses

Total RNA was extracted using TRIzol^®^ Reagent. First-strand cDNA was synthesized using a PrimeScript^®^ RT Reagent Kit (TAKARA). Synthesized cDNA was subjected to RT-PCR using Premix Ex Taq™ Version 2.0 (TAKARA) with specific primers. Moreover, qPCR was performed with SYBR^®^ Premix Ex Taq™ (TAKARA) on CFX96 (Bio-Rad) according to the manufacturer’s protocol. The primers for qPCR are listed in Supplementary Table S2.

### Bisulfite sequencing

The bisulfite treatment of genome DNA was performed using the EZ-DNA methylation direct kit (Zymo Research) according to the manufacturer’s instructions. The primer sequences for Oct4 and Elf5 are listed in Supplementary Table S2 supplemental material. Amplified products were cloned into pMD18T vector (TAKARA), and sequenced using M13F primers. CpG methylation was analyzed using Alignment software. For each promoter sequence, 6–8 clones were sequenced.

### Immunocytochemistry

Cells were fixed using 4% paraformaldehyde (PFA, Sigma), washed three times with phosphate buffer solution (PBS) and incubated for 45 minutes in blocking buffer (5% goat serum, 1% BSA, and 0.2% Triton X-100 in PBS) at room temperature (RT). After removing the blocking buffer, the cells were incubated overnight with primary antibody at 4 °C. The primary antibodies used were Mouse anti-Oct4 (Santa Cruz, 1:100), rabbit anti-CDX2-88 (BioGenex, 1:1), rabbit anti-EOMES (Millipore, 1:200), goat anti-Sox17 (RD, 1:200), and goat anti-Hnf3b (Santa Cruz, 1:100). The next day, the slides were washed with PBS and added with fluorescence-labeled secondary antibody in the dark at RT for 1 h. The cells were subsequently washed with PBS and stained with DAPI solution (1 μg/mL).

### *In vitro* differentiation assay

TSCs or cviTSCs were cultured in TS-differentiation medium: RPMI1640 supplemented with 20% FBS, 1% L-Glutamine, 1% sodium pyruvate, and 0.1 mM β-mercaptoethanol. The medium was refreshed every other day. After 6 days, differentiated cells were stained by Hochest33342 (1 μg/mL, Sigma) to determine the number of nucleus.

### DNA content assay

cviTSCs were differentiated as described above. Cells were collected at selected time points, dissociated in trypsin-EDTA (0.05%, Life Technologies), and fixed with 75% ice-cold ethanol. Cells were stained in 4′,6-diamidino-2-phenylindole (DAPI) staining solution with 1 μg/mL DAPI (Sigma), 0.1% (v/v) Triton X-100 (Sigma), and 0.2 mg/mL RNaseA (Sigma) in PBS for 10 min at RT. Flow cytometry was performed on a FACSCanto (BD Biosciences) and analyzed using FlowJo Software (Tree Star).

### Invasion assay *in vitro*

Polycarbonate membrane transwell chambers with an 8 mm pore size (Corning) were coated with Matrigel (1:25, BD) in DMEM/F12 medium overnight and rehydrated with DMEM medium the next day for 2 h at 37 °C under 5% CO2. TSCs were plated on top of the chambers in TS-L medium: 1640 medium supplemented with 1% BSA fraction V (life technologies). Low chamber was added with TS-H medium: 1640 medium supplemented with 20% FBS. After 3 days, membranes were fixed in 4% PFA followed PBS treatment with 0.2% Triton X-100 and 0.4% Typan Blue for 40 min. The cells on top of each membrane were scraped off. Cells on the transwell membranes were counted under a microscope (Olympus X51).

### cviTSC subcutaneous injection

cviTSCs were labeled with green fluorescence as previous report^43^. Briefly, FUGW were packaged by co-transfecting 293T cells with auxiliary packaging vectors psPAX2 and pMD2-G. Lentiviruses were harvested after 48 h and added to TSC culture medium. The MOI (MOI = viral titer/cell number) is about 30 ~ 50. A total of 1 × 10^6^ EGFP-cviTSCs were collected and resuspended in 200 μL of TSC-X medium containing 20% Matrigel (BD). Cells were subcutaneously injected into male ICR mice, which were immunosuppressed with cyclosporine A (10 mg/kg) each day. After 6 days, the mice were killed by cervical dislocation. Tumors were dissected, fixed in 4% formalin, and routinely embedded in paraffin for immunohistochemistry.

### Chimera assay

After culturing for 15 passages, EGFP-cviTSCs were collected. For 4-cell-embryo injection, these cells were injected into ICR 4-cell-embryo with 5–8 cells. The embryos were cultured *in vitro* until the blastocyst stage. For blastocyst injection, EGFP-cviTSCs were microinjected into ICR blastocysts with 9–15 cells in one blastocyst. Blastocysts were transferred into pseudopregnant ICR females. After 9–10 days, the embryos and placentas were separated from uterus. The injected cells were traced under fluorescent a microscope (Olympus SZX16).

cviXENCs were labeled with red fluorescence by transfecting with vector FUW-DsRed. The process for blastocyst injection was the same with that described above. After transplanting them into pseudopregnant mice for 4 and 5 days, embryos were collected and observed under fluorescent microscopes (Olympus SZX16 and Olympus X51).

### Immunohistochemistry

Blood-filled sinuses, placentas and embryos were fixed *in situ* with 4% buffered PFA for 48 h. The entire tissue was then dissected, embedded in paraffin wax, and exhaustively cross-sectioned at 3 μM to a slide. Sections were deparaffinized in xylene and rehydrated in a graded series of alcohol, followed by dH2O. Antigen retrieval was performed in a 1 M citrate buffer (pH = 6.0) bath for 20 min. Sections were incubated in 3% H2O2 for 10 min followed by 5% goat serum for 1 h at room temperature to reduce nonspecific staining. Tissues were incubated with primary antibody anti-EGFP (1:200, Sigma) and Cytokeratin 8 (KRT8, 1:100, Proteintech) at 4 °C overnight followed by incubation with secondary antibody (1:1000, ZSGB-BIO) for 1 h at room temperature. Finally, samples were visualized using diaminobenzidine (DAB) kit (ZSGB-BIO) and the nuclei were stained with hematoxylin (Sigma).

### Statistical analysis

Unpaired two-tailed Student’s t-test were used to assess statistical significance. Statistical analysis was done by GraphPad Prism software (Version 6.0 for Windows. GraphPad Software, San Diego, CA). p value < 0.05 was considered as statistically significant, ^*^p < 0.05, ^**^p < 0.01, ^***^p < 0.001. Data are presented as mean ± s.d. as indicated in the figure legends. All sample numbers listed indicate the number of biological replicates employed in each experiment.

## Additional Information

**How to cite this article**: Wei, S. *et al.* Conversion of embryonic stem cells into extraembryonic lineages by CRISPR-mediated activators. *Sci. Rep.* **6**, 19648; doi: 10.1038/srep19648 (2016).

## Supplementary Material

Supplementary Information

## References

[b1] Takahashi K. *et al.* Induction of pluripotent stem cells from adult human fibroblasts by defined factors. Cell 131, 861–872 (2007). 18035408 10.1016/j.cell.2007.11.019

[b2] Takahashi K. & Yamanaka S. Induction of Pluripotent Stem Cells from Mouse Embryonic and Adult Fibroblast Cultures by Defined Factors. Cell 126, 663–676 (2006). 16904174 10.1016/j.cell.2006.07.024

[b3] Vierbuchen T. *et al.* Direct conversion of fibroblasts to functional neurons by defined factors. Nature 463, 1035–1041 (2010). 20107439 10.1038/nature08797 PMC2829121

[b4] Pang Z. P. *et al.* Induction of human neuronal cells by defined transcription factors. Nature 476, 220–223 (2011). 21617644 10.1038/nature10202 PMC3159048

[b5] Marro S. *et al.* Direct lineage conversion of terminally differentiated hepatocytes to functional neurons. Cell Stem Cell 9, 374–382 (2011). 21962918 10.1016/j.stem.2011.09.002 PMC3218088

[b6] Huang P. *et al.* Induction of functional hepatocyte-like cells from mouse fibroblasts by defined factors. Nature 475, 386–389 (2011). 21562492 10.1038/nature10116

[b7] Sekiya S. & Suzuki A. Direct conversion of mouse fibroblasts to hepatocyte-like cells by defined factors. Nature 475 390–393 (2011). 21716291 10.1038/nature10263

[b8] Jinek M. *et al.* A programmable dual-RNA-guided DNA endonuclease in adaptive bacterial immunity. Science 337, 816–821 (2012). 22745249 10.1126/science.1225829 PMC6286148

[b9] Cong L. *et al.* Multiplex genome engineering using CRISPR/Cas systems. Science 339, 819–823 (2013). 23287718 10.1126/science.1231143 PMC3795411

[b10] Mali P. *et al.* RNA-guided human genome engineering via Cas9. Science 339, 823–826 (2013). 23287722 10.1126/science.1232033 PMC3712628

[b11] Bikard D. *et al.* Exploiting CRISPR-Cas nucleases to produce sequence-specific antimicrobials. Nat biotechnol 32, 1146–1150 (2014). 25282355 10.1038/nbt.3043 PMC4317352

[b12] Maeder M. L. *et al.* CRISPR RNA-guided activation of endogenous human genes. Nat methods 10, 977–979 (2013). 23892898 10.1038/nmeth.2598 PMC3794058

[b13] Perez-Pinera P. *et al.* RNA-guided gene activation by CRISPR-Cas9-based transcription factors. Nat methods 10, 973–976 (2013). 23892895 10.1038/nmeth.2600 PMC3911785

[b14] Gilbert L. A. *et al.* CRISPR-mediated modular RNA-guided regulation of transcription in eukaryotes. Cell 154, 442–451 (2013). 23849981 10.1016/j.cell.2013.06.044 PMC3770145

[b15] Artus J. & Chazaud C. A close look at the mammalian blastocyst: epiblast and primitive endoderm formation. Cell Mol Life Sci 71, 3327–3338 (2014). 24794628 10.1007/s00018-014-1630-3 PMC11113690

[b16] Cho L. T. *et al.* Conversion from mouse embryonic to extra-embryonic endoderm stem cells reveals distinct differentiation capacities of pluripotent stem cell states. Development 139, 2866–2877 (2012). 22791892 10.1242/dev.078519 PMC3403099

[b17] Niwa H. *et al.* Interaction between Oct3/4 and Cdx2 determines trophectoderm differentiation. Cell 123, 917–929 (2005). 16325584 10.1016/j.cell.2005.08.040

[b18] Shimosato D., Shiki M. & Niwa H. Extra-embryonic endoderm cells derived from ES cells induced by GATA factors acquire the character of XEN cells. BMC Dev Biol 7, 80 (2007). 17605826 10.1186/1471-213X-7-80 PMC1933422

[b19] Gao X. *et al.* Reprogramming to Pluripotency Using Designer TALE Transcription Factors Targeting Enhancers. Stem Cell Rep 1, 183–197 (2013). 10.1016/j.stemcr.2013.06.002 PMC3757749 24052952

[b20] Kubaczka C. *et al.* Derivation and Maintenance of Murine Trophoblast Stem Cells under Defined Conditions. Stem Cell Rep 2, 232–242 (2014). 10.1016/j.stemcr.2013.12.013 PMC3923226 24527396

[b21] Roberts R. M. & Fisher S. J. Trophoblast stem cells. Biol Reprod 84, 412–421 (2011). 21106963 10.1095/biolreprod.110.088724 PMC3043125

[b22] Kuckenberg P. *et al.* Lineage conversion of murine extraembryonic trophoblast stem cells to pluripotent stem cells. Mol Cell Biol 31, 1748–1756 (2011). 21300784 10.1128/MCB.01047-10 PMC3126346

[b23] Parast M. M. *et al.* PPARgamma regulates trophoblast proliferation and promotes labyrinthine trilineage differentiation. PloS One 4, e8055 (2009). 19956639 10.1371/journal.pone.0008055 PMC2778869

[b24] Hemberger M., Hughes M. & Cross J. C. Trophoblast stem cells differentiate *in vitro* into invasive trophoblast giant cells. Dev Biol 271, 362–371 (2004). 15223340 10.1016/j.ydbio.2004.03.040

[b25] Rossant J. & Cross J. C. Placental development: lessons from mouse mutants. Nat Rev Genet 2, 538–548 (2001). 11433360 10.1038/35080570

[b26] Tanaka S., Kunath T., Hadjantonakis A. K., Nagy A. & Rossant J. Promotion of trophoblast stem cell proliferation by FGF4. Science 282, 2072–2075 (1998). 9851926 10.1126/science.282.5396.2072

[b27] Murray P. & Edgar D. Regulation of the differentiation and behaviour of extra-embryonic endodermal cells by basement membranes. J Cell Sci 114, 931–939 (2001). 11181176 10.1242/jcs.114.5.931

[b28] Chakraborty S. *et al.* A CRISPR/Cas9-Based System for Reprogramming Cell Lineage Specification. Stem Cell Rep 3, 940–947 (2014). 10.1016/j.stemcr.2014.09.013 PMC4264059 25448066

[b29] Chavez A. *et al.* Highly efficient Cas9-mediated transcriptional programming. Nat methods (2015). 10.1038/nmeth.3312 PMC4393883 25730490

[b30] Gilbert L. A. *et al.* Genome-Scale CRISPR-Mediated Control of Gene Repression and Activation. Cell 159, 647–661 (2014). 25307932 10.1016/j.cell.2014.09.029 PMC4253859

[b31] Nichols J. *et al.* Formation of pluripotent stem cells in the mammalian embryo depends on the POU transcription factor Oct4. Cell 95, 379–391 (1998). 9814708 10.1016/s0092-8674(00)81769-9

[b32] Wu T. *et al.* Reprogramming of trophoblast stem cells into pluripotent stem cells by Oct4. Stem Cells 29, 755–763 (2011). 21305674 10.1002/stem.617

[b33] Gao X. *et al.* Comparison of TALE designer transcription factors and the CRISPR/dCas9 in regulation of gene expression by targeting enhancers. Nucleic Acids Res 42, e155 (2014). 25223790 10.1093/nar/gku836 PMC4227760

[b34] Morrisey E. E., Ip H. S., Lu M. M. & Parmacek M. S. GATA-6: a zinc finger transcription factor that is expressed in multiple cell lineages derived from lateral mesoderm. Dev Biol 177, 309–322 (1996). 8660897 10.1006/dbio.1996.0165

[b35] Niakan K. K. *et al.* Sox17 promotes differentiation in mouse embryonic stem cells by directly regulating extraembryonic gene expression and indirectly antagonizing self-renewal. Gene Dev 24, 312–326 (2010). 20123909 10.1101/gad.1833510 PMC2811832

[b36] Ji Q. *et al.* Engineered zinc-finger transcription factors activate OCT4 (POU5F1), SOX2, KLF4, c-MYC (MYC) and miR302/367. Nucleic Acids Res 42, 6158–6167 (2014). 24792165 10.1093/nar/gku243 PMC4041418

[b37] Hu J. *et al.* Direct activation of human and mouse Oct4 genes using engineered TALE and Cas9 transcription factors. Nucleic Acids Res 42, 4375–4390 (2014). 24500196 10.1093/nar/gku109 PMC3985678

[b38] Hilton I. B. *et al.* Epigenome editing by a CRISPR-Cas9-based acetyltransferase activates genes from promoters and enhancers. Nat Biotechnol 33, 510–517 (2015). 25849900 10.1038/nbt.3199 PMC4430400

[b39] Huangfu D. W. *et al.* Induction of pluripotent stem cells by defined factors is greatly improved by small-molecule compounds. Nat Biotechnol 26, 795–797 (2008). 18568017 10.1038/nbt1418 PMC6334647

[b40] Niakan K. K., Schrode N., Cho L. T. & Hadjantonakis A. K. Derivation of extraembryonic endoderm stem (XEN) cells from mouse embryos and embryonic stem cells. Nat Protoc 8, 1028–1041 (2013). 23640167 10.1038/nprot.2013.049 PMC3927835

[b41] Mali P. *et al.* CAS9 transcriptional activators for target specificity screening and paired nickases for cooperative genome engineering. Nat Biotechnol 31, 833–838 (2013). 23907171 10.1038/nbt.2675 PMC3818127

[b42] Li Z. *et al.* Mouse SCNT ESCs have lower somatic mutation load than syngeneic iPSCs. Stem Cell Rep 2, 399–405 (2014). 10.1016/j.stemcr.2014.02.005 PMC3986627 24749065

[b43] Zou Q. *et al.* Direct conversion of human fibroblasts into neuronal restricted progenitors. J Biol Chem 289, 5250–5260 (2014). 24385434 10.1074/jbc.M113.516112 PMC3931081

