# Supplementary material for: Conversion of embryonic stem cells into extraembryonic lineages by CRISPR-mediated activators
Source: Sci Rep. 2016 Jan 19;6:19648. doi: 10.1038/srep19648 (PMC4726097; doi:10.1038/srep19648)
Supplement: Supplementary Information [file srep19648-s1.pdf]

Supplementing Information

Conversion of embryonic stem cells into extraembryonic lineages by CRISPR-mediated activators

Shu Wei, Qingjian Zou, Sisi Lai, Quanjun Zhang, Li Li, Quanmei Yan, Xiaoqing Zhou, Huilin Zhong, Liangxue Lai

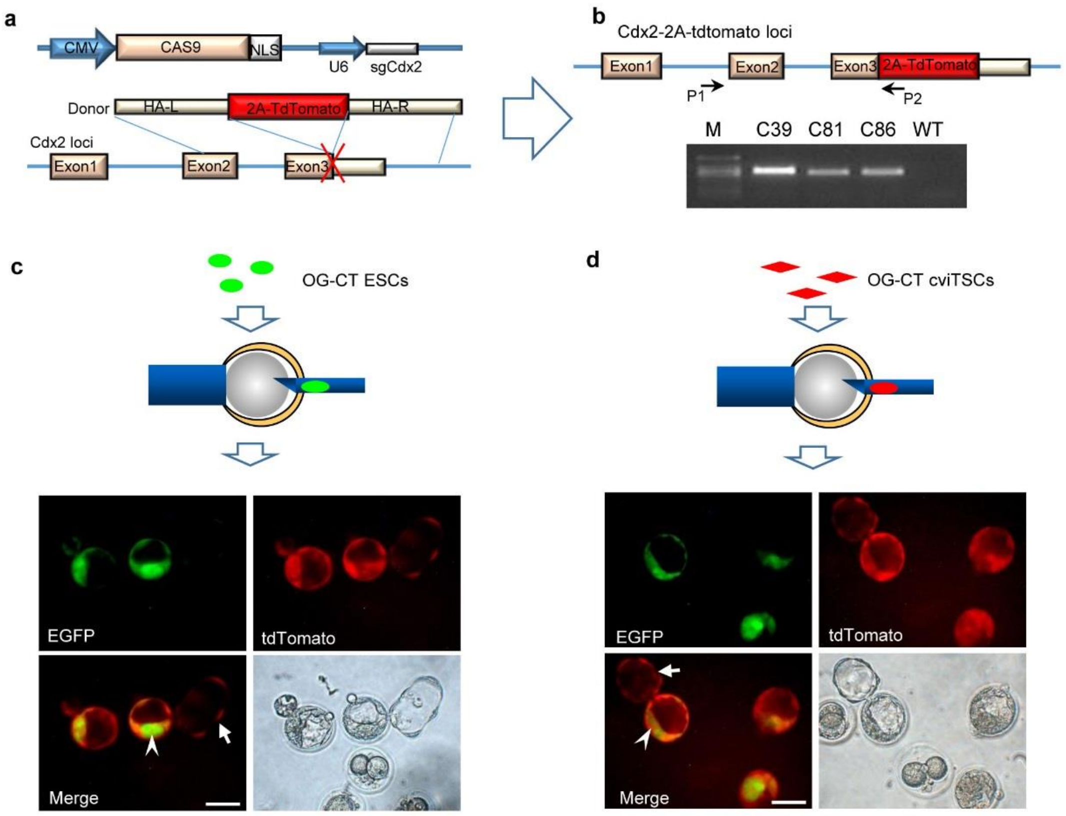

Supplementary Figure 1. Generation and identification of Cdx2 reporter system. (**a**) Schematic overview of strategy to generate a Cdx2-2A-tdTomato knock-in allele. A 2A-tdTomato cassette was site-specific integrated adjacently before the stop codon of Cdx2. The homologous arms of the donor vector are indicated as HA-L (1.6 kb) and HA-R (1.4 kb). Black arrows indicate the primers for knock-in identification. (**b**) Genomic PCR for 2A-tdTomato knock-in positive ES colonies (OG-CT ESCs). M, marker; C, colonies; WT, wild type. (**c**) The OG-CT ESCs were transferred into recipient oocytes and developed to the blastocyst stage *in vitro*. Cells with green fluorescence in ICMs (arrowhead) and red fluorescence in TEs (arrow) were visualized by microscopy. Scale bar, 100 µm. (**d**) Reprogramming of cviTSCs by nuclear transfer. cviTSCs were transferred into enucleated oocytes and developed into the blastocyst stage *in vitro*. Cells with green fluorescence in ICMs (arrowhead) and red fluorescence in TEs (arrow) were visualized by microscopy. Scale bar, 100 µm.

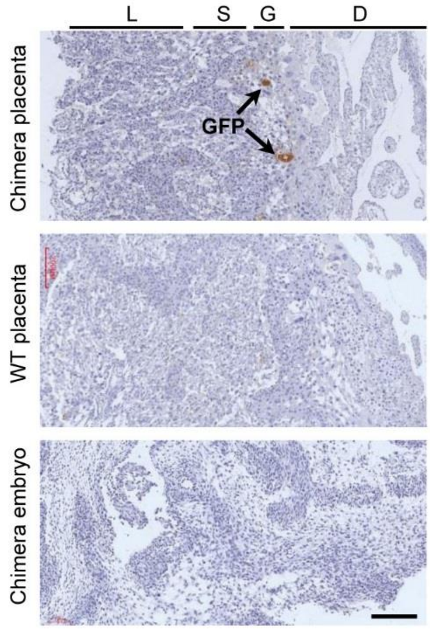

Supplementary Figure 2. The specificity of EGFP. GFP positive cells derived from cviTSCs embedded in chimera placenta, but not in WT placenta and chimera embryo. WT, wild type; staining D, decidua; G, giant cell; S, spongiotrophoblast; L, labyrinth. Scare bar= 200 um.

Supplementary Table 1. sgRNA target site sequences and locations.

Targets	20nt sequence (5’ to 3’)	PAM	Strand	Location (TSS=0)
Cdx2P-g1	GGACTCCGCGAGCCAACCTG	CGG	+	+19
Cdx2P-g2	GCCTCGACGTCTCCAGCCAT	TGG	+	−68
Cdx2P-g3	GCAAATCGTGTTTCTGGGGG	TGG	+	−122
Cdx2P-g4	GTCTTTGAACCTGTGATTGG	AGG	+	−262
Cdx2P-g5	GGGAAGCTGGGTTTGGAAAC	GGG	−	−338
Cdx2P-g6	GGAAATAAATGTATTGCTGA	GGG	+	−584
Gata6P-g1	GAGAAGGAGGAGGGATCCAG	GGG	−	−41
Gata6P-g2	GCAGCTCAACCCACGCCCGC	AGG	+	−144

Supplementary Table 2. primers for RT-PCR, qPCR and Bisulfite PCR.

Gene	Primer sequences
Gapdh	F: CGACTTCAACAGCAACTCCCACTCTTCC
	R: TGGGTGGTCCAGGGTTTCTTACTCCTT
Oct4	F: AATGCCGTGAAGTTGGAGAAGG
	R: CAAAGAGAACGCCCAGGGTGAG
Tcfap2c	F: CTTACGCTGCCGCCATGAAC
	R: CCCCTCCAGCCCTGAAATATG
Eomes	F: CGGGTCTTGTGGAGGATTGGG
	R: GGTGGGGTTGAGTCCGTTTATG
Elf5	F: AACAAGCCTCCAAAGTTCTCACC
	R: CTTAACCACTCGGAAAATGCCCT
Endo-Cdx2	F: AGGAGTATGGACGCTGCGAGAATC
	R: ATCTAAACCATGTCTTCCCCTGAGG
Tead4	F: GATCCCGACACATACAACAAACACC
	R: CACAAGGAAGAAGGCATTAGAGGG
Nanog	F: CCCTGATTCTTCTACCAGTCCCAAAC
	R: GCTTCTGAAACCTGTCCTTGAGTGC
Sox2	F: TGCGCCCAGTAGACTGCACATG
	R: AAAATCTCTCCCCTTCTCCAGTTCG
Gcm1	F: GGCAAGAGCAACAGGTGGGA
	R: CAAATCACGACTGGGAGAGAGAAGG
Tpbp	F: GCACAGCTTTGGACATCACAGGTAC
	R: GAACTTCTTTATCCTTCTGCTCTTGC
Pl1	F: GCTGCTGGTGTCAAGCCTACTCC
	R: AAATTCCCTATACACATCTGCGGC
Pl2	F: CAAGAACAAAGGAGTTGGAGGAAAG
	R: CGCCATAAGGTTCTAAGAGCACTG
Plf	F: AATGGTCGTTGCTTTATGTCCTTTG
	R: GGAGTTGGAAGGAAAGAAGTATTGC
Oct4-meth	F: TGGGTTGAAATATTGGGTTTATTT
	R: CCCCCTGGGTAAATAAAAAAACAAAAC
Elf5-meth	F: GTGGAAAGGTTAGTGAAAGTATTG
	R: AATTTATACACAACCCCTTTAATAAC
Endo-Gata6	F: GAGCTGGTGCTACCAAGAGG
	R: TGCAAAAGCCCATCTCTTCT
Gata4	F: CCCTTCCCTCTTCAAATTCC
	R: CTTTTCCAGAGCTCCACCTG
Sox17	F: GAGGGCCAGAAGCAGTGTTA
	R: AGTGATTGTGGGGAGCAAGT
Sox7	F: GCTCCTGCTTTTGGTGTAGC
	R: GTCCTTGGGCAGTCATTCAT
Pthr1	F: AGGACGACGGCTTCCTTAAT

	R: TTGTCTTCCTGGTCCAGTCC
Sparc	F: GTTCCTGCTTGGCTCTCTTG
	R: CCTTGAGGGAGGTAGGGAAG
Afp	F: TCCAGAAGGAAGAGTGGACAA
	R: GCAGACTAGGAGAAGAGAAATAGTTGA
Ihh	F: GGCCTGGGATTGTGACTTTA
	R: CTGCAGGGAAGGTCATGTTT
